# Wildflower strips enhance pollination in adjacent strawberry crops at the small scale

**DOI:** 10.1002/ece3.4631

**Published:** 2018-11-06

**Authors:** Dominik Ganser, Barbara Mayr, Matthias Albrecht, Eva Knop

**Affiliations:** ^1^ Institute of Ecology and Evolution University of Bern Bern Switzerland; ^2^ Agroscope, Agroecology and Environment Zürich Switzerland

**Keywords:** agri‐environment schemes, ecosystem services, mass‐flowering crops, pollination services, strawberry, wild bees, wildflower strips

## Abstract

Wildflower strips (WFS) are increasingly used to counteract the negative consequences of agricultural intensification. To date, it is poorly understood how WFS promote flower visitation and pollination services in nearby insect‐pollinated crops. We therefore ask whether WFS enhance pollination service in adjacent strawberry crops, and how such an effect depends on the distance from WFS. Over 2 years, we examined the effects of experimentally sown WFS compared to grassy strips on pollination services in adjacent strawberry (*Fragaria ananassa*) crops across a total of 19 study sites. Moreover, we examined flower visitation, species richness and community composition of the most important insect pollinator taxa at different within‐field locations varying in distance to WFS. We found increased pollination services at the edge of WFS compared to locally reduced pollination services at the center, which resulted in no significant difference in seed set between WFS and control fields. Total flower visits and species richness of pollinators were higher in WFS than in adjacent strawberry fields. Moreover, wild bee visitation was enhanced in adjacent strawberry crops near WFS compared to field centers, and intermediate at field edges near grassy strips. Our study demonstrates that diverse WFS can increase wild bee visitation and pollination services in the field edges of adjacent strawberry crops, but that overall visitation and pollination services do not increase. Moreover, our findings show that major pollinator taxa exhibit distinct responses, resulting in a shift of pollinator community composition as a function of distance to WFS with direct effects on crop pollination. Our results that WFS enhance rather than reduce crop pollination services near WFS should distract possible concerns by farmers that WFS may locally absorb rather than export crop pollinators. Considering the spatial restricted enhancement of wild bees and associated pollination services we suggest to establish WFS in the center of crop fields.

## 1 INTRODUCTION

Managed and wild pollinators provide important crop pollination services and can thereby improve the yield of many animal‐pollinated crops (IPBES, 2016). In fact, insect‐mediated pollination can increase yields in an estimated 75% of the world's leading crops (Klein et al., 2007). Managed honey bees (*Apis mellifera* L.) are used widely as crop pollinators, but recent global meta‐analyses have highlighted the importance of wild pollinators for crop pollination (e.g. Garibaldi et al., 2014; Holzschuh, Dudenhöffer, & Tscharntke, 2012; Mallinger & Gratton, 2015; Winfree, Williams, Dushoff, & Kremen, 2007), which account for roughly half of the economic value of pollination services worldwide (Kleijn et al., 2015).

Recent declines in managed and wild pollinators in regions of North America and Europe (Biesmeijer, 2006; Cameron et al., 2011; IPBES, 2016) jeopardize pollinator diversity as well as the delivery of pollination services to wild plants and crops, potentially resulting in yield deficits of crops (Aizen & Harder, 2009; Cusser, Neff, & Jha, 2016; IPBES, 2016). In agroecosystems, pollinator decline has been mainly attributed to habitat loss and degradation due to intensification of agricultural practices, introduced pathogens and parasites, pesticide exposure and climate change, and the interactions of these drivers (Desneux, Decourtye, & Delpuech, 2007; Goulson, Nicholls, Botias, & Rotheray, 2015; Winfree & Kremen, 2009).

The loss and degradation of semi‐natural habitats has reduced the amount of floral resources (Goulson, Lye, & Darvill, 2008; Williams & Osborne, 2009) and the availability of nesting sites for pollinators (Steffan‐Dewenter & Schiele, 2008), which are considered the underlying mechanism of wild pollinator decline in agroecosystems (e.g. IPBES, 2016). Although mass‐flowering crops are offering rewarding floral resources in intensive agricultural landscapes, they are ephemeral and only available during short time periods, and often not congruent with the foraging periods of many wild bee species (Westphal, Steffan‐Dewenter, & Tscharntke, 2006). In fact, not only is the amount of available floral resources important for pollinators, but also its continuous availability and phenological completeness. Different pollinator taxa differ in their activity periods throughout the season and therefore need floral resources at different times. On the other hand, pollinators with long lifecycles, such as bumble bees or honey bees, are critically affected in their health (Alaux et al., 2017), reproduction success (Williams, Regetz, & Kremen, 2015; Williams, Ward, et al., 2015) and the survival between lifecycle stages (Carvell et al., 2017) without enough food resources throughout the entire activity periods.

Species‐rich wildflower strips (WFS) are increasingly used to provide a diversity of floral resources across the entire flowering season to mitigate the negative consequences of agricultural intensification on pollinators. Moreover, the aim of most of these floral enhancement measures in agroecosystems is to concomitantly promote crop pollination services (Haaland, Naisbit, & Bersier, 2011; Scheper et al., 2013). In Europe, WFS are often promoted as part of agri‐environmental schemes (Carvell, Bourke, Osborne, & Heard, 2015). While many studies have addressed their potential contribution to the conservation of farmland biodiversity, including pollinators (reviewed e.g. in Haaland et al., 2011; Scheper et al., 2013), much less is known about potential spillover of different pollinator taxa into adjacent crops, and the consequences of WFS establishment on the delivery of pollination services in nearby insect‐pollinated crops (but see Balzan, Bocci, & Moonen, 2016; Blaauw & Isaacs, 2014; Sutter, Jeanneret, Bartual, Bocci, & Albrecht, 2017; Venturini, Drummond, Hoshide, Dibble, & Stack, 2017).

Although the few studies available suggest that WFS may enhance rather than reduce pollination services in nearby crops, current evidence is not conclusive and mechanisms behind the observed effect are less clear. Therefore, another possibility is under debate: WFS, through the provision of high amounts of pollen and nectar resources, may locally concentrate rather than export crop pollinators. This hypothesis assumes that during the flowering period of insect‐pollinated crops, the presence of WFS may, at least temporary, lead to local competition for crop pollinators, and consequently reduced flower visitation and pollination in nearby crops (Lander, Bebber, Choy, Harris, & Boshier, 2011). This is a major concern of many farmers (Garbach & Long, 2017), hampering the implementation of WSF and other types of floral enhancement measures to restore pollinator and other flower‐visiting insect communities in agroecosystems. Whether pollinators concentrate in WFS or spillover into nearby crops may differ among taxonomic and life‐history groups of pollinators. Moreover, the spatial scale over which concentration or spillover affect the distribution of pollinators in crop fields may strongly vary among major pollinator groups, with potentially important consequences on the delivery of pollination services and crop yield as a function of distance to WFS.

Here, we therefore asked whether (a) WFS enhance pollination service provisioning in adjacent strawberry crops; how (b) such an effect depends on the distance from WFS (field edge near WFS or field center); and (c) which of the major pollinator groups (honey bees, bumble bees, other wild bees and hover flies) might cause such an effect. In particular, do all WFS enhance visitation rates to strawberry crops of all major pollinator groups equally or are there groups of pollinators which less likely disperse into the adjacent field.

## 2 MATERIALS AND METHODS

### 2.1 Study design

A total of 19 commercial strawberry fields were randomly chosen in the central Swiss plateau (34 × 133 km, cantons Bern, Zurich, Solothurn, and Aargau). The area is characterized as a typical Swiss agricultural landscape consisting of arable crops, grasslands, and forests in a relatively small‐scaled mosaic. The minimum distance between fields was 3 km. Along a randomly chosen border of each of 12 strawberry fields, three different mixtures of WFS were sown. The remaining seven strawberry fields contained an adjacent linear, regularly mown grassy strip instead of a WFS and served as control fields. Field size, size of the field‐bordering linear vegetation (flower or grassy strip), landscape complexity and composition surrounding WFS fields were similar compared to control fields (Supporting Information Table S1). WFS were sown in September 2015 (mixture 1; six farms) and March 2016 (mixture 2; six farms). Farms which contained WFS with mixture 1 were re‐sown in September 2016, from which three WFS had to be abandoned due to weed problems (mixture 3, three farms; see Supporting Information Table S1). The mixtures consisted of annual and perennial (mixture 2) wildflower species, which were selected based on fast‐growing and high pollen and nectar rewards for bees, hover flies and other flower‐visiting insects (see Supporting Information Table S2 for a detailed description of seed mixtures). WFS did not significantly differ in species richness (ANOVA: *F*_2,12_, *p = *0.692) or flower abundance during the sampling period (ANOVA: *F*_2,12_, *p* = 0.583, Supporting Information Table S4). Average species richness of flowering plants in WFS was 8.12 ± 2.34 (mean ± *SE* throughout). Each WFS had a width of 6 m, but varied in length with a minimum of 80 m (0.099 ± 0.03 ha). Of all strawberry fields, the border opposite to the WFS or control strip was a grassy, regularly mowed strip with a minimum width of 5 m. None of the strawberry producers used managed honey bees or other commercially available bees (e.g. *Bombus terrestris* or *Osmia bicornis*) to support pollination of strawberry of the selected fields, and there were no honey bee hives in the direct vicinity of the focal fields (within a 1 km radius). Nevertheless, social bees like honey bees or bumble bees are able to cover forage flight distances of several kilometers (Greenleaf, Williams, Winfree, & Kremen, 2007) and were abundant within the study sites.

### 2.2 Seed set measurements

In two subsequent years (2016, 2017), potted strawberry plants (early blooming variety “Lambada”; 360 plants) were grown in the green house for 3 weeks. Strawberry are highly dependent on the pollination for marketable fruit although they are self‐fertile (e.g. Klatt et al., 2014). They are regularly visited by pollinating insects, which includes bees, wasps, beetles, flies, ants, and butterflies (Albano, Salvado, Duarte, Mexia, & Borges, 2009). Thus, with the beginning of flowering (end of May) strawberry plants were brought into focal fields (2016: 12 WFS fields, seven control fields; 2017: 9 WFS fields, seven control fields) and left there until all flowers were withered (end of June). All open flowers were removed before bringing the plants into focal fields. All focal fields adjacent to WFS were equipped with 30 potted plants (see Supporting Information Figure S1 for a detailed description of the study plan). Ten plants were placed at the crop edge next to the WFS with a minimum distance of 2 m to the WFS. Additionally, ten plants were placed in the center and ten plants on the opposite side of the strawberry field edging grassy strips (crop edge other). Due to different sizes of the strawberry fields, this resulted in varying distances of pots at the crop edge grassy location to the WFS (35–160 m). To prevent strawberry plants from water stress, each pot was watered regularly. In 2016, we additionally equipped the plants with a watering system adapted from Turrini and Knop (2015). Control fields without adjacent WFS were equipped with 20 plants: 10 at the center of the field (control center) and 10 at the edge adjacent to a grassy strip (control edge). After blooming was over plants were brought to the greenhouse again. Fully developed fruits were harvested and frozen for further seed set analysis. To determine seed set (proportion of fertilized seeds per fruit), fruits were filtered through a fine sieve with a mesh size of 0.1 mm to separate seeds from other fruit parts. Subsequently, seeds were put into a bucket filled with water, where the lighter unfertilized seeds swum at the water surface and heavier fertilized seeds sank to the bottom (Klatt et al., 2014).

### 2.3 Sampling of flower visitation by bees and hover flies

The abundance and species richness of the major groups of flower‐visiting insects (managed western honey bees (*A. mellifera* L.), bumble bees (*Bombus* spp.), other wild bees, and hover flies) were only assessed in 2017 and only on the WFS and the adjacent strawberry field (see Supporting Information Table S3 for a species list of pollinators). Sampling was conducted on each site (WFS and adjacent strawberry field) three times between May and June, which corresponded to the peak bloom of the strawberries. To examine flower visitation pollinators were collected along four distinct belt transects of 80 m length and a width of 2 m (see below): one transect in the center of the WFS and three parallel transects varying in their distance to the WFS (2–160 m). One of the three transects was at the edge of strawberry fields next to the WFS (crop edge WFS), in the center of the strawberry field (crop center) and at the opposite field edge of the field (crop edge other). Belt transects were walked at a slow pace, recording all pollinator visits to the reproductive structure of flowers during a maximum of 20 min per transect. Time was stopped for the duration of insect handling. Surveys were conducted between 10:00 and 16:00 on sunny days with temperatures above 16°C. Flower visitors that could not be identified in the field were collected for later identification. Flower visits of pollinators were standardized for the different field sizes of WFS and strawberry crop. Additionally, flower abundance was estimated as the total number of open flowers present per 20 m^2^ plot. The numbers of open flowers were counted by species in five 4 m^2^ quadrats randomly selected within the WFS. In strawberry fields, the number of open strawberry flowers was counted per 1 m × 80 m transect and divided by 4. The number of strawberry flowers available did not differ between sampled locations within strawberry fields (*p* = 0.537, Table S4). To standardize flower abundance the floral area was defined using methods described in Williams, Ward, et al. (2015) and Williams, Regetz, et al. (2015). Diameter or length and width of flowers per species were measured once a season using five inflorescences per species.

### 2.4 Statistical analyses

To examine the effect of WFS (strawberry crop fields with or without adjacent WFS, question a) and whether such an effect depends on the distance from the WFS (question b), we run a generalized linear mixed‐effects (GLMM) model assuming a binomial distribution. The model included the ratio of fertilized seeds as dependent variable, the management of the edge of the crop field (management, two levels: strawberry crop fields with adjacent WFS vs. strawberry crop fields without adjacent WFS) and the location of the potted strawberry plants within strawberry fields as fixed factor (location, two levels: edge, center), and plant (10 per site) nested within site (19 sites) nested within year (2016, 2017) as a random factor. In addition, the interaction management:location was included to analyze whether a potential edge effect differs between fields with WFS and fields without WFS. All GLMM models were checked for overdispersion by including an observation‐level random factor (levels of observations) into the model and comparing it to a model without the random observation parameter. In case the observation‐level random factor significantly improved the model, it was remained in the model.

To analyze whether the number of flower visitations and number of species of major pollinator groups (honey bees, bumble bees, other wild bees and hover flies) is increased on WFS compared to crop sites, and which groups of pollinators disperse into crop sites, we run GLMM models with a Poisson distribution for each of the major pollinator groups separately. The models included the number of flower visitations and number of species of pollinators as dependent variable and the location within the study site (factor location with four levels, WFS, crop edge WFS, crop center, crop edge other) as a fixed factor. To analyze, whether the effect of WFS is mainly due to increased floral abundance or whether there is an additional effect of the WFS, we run the same model but also included flower units as explanatory variable. Round (three levels) nested in study site (nine sites) was included in both models as a random factor. For all models, we checked beforehand whether effects varied based on the seed mixtures, which was not the case. Additionally, Tukey's post hoc tests for multiple comparisons were used to disentangle the effects of the different edge positions in the field (crop edge WFS vs. crop edge other) for the different pollinator groups using the multcomp package (R package multcomp version 1.3–6; Hothorn et al., 2014). All analyses were done in R version 3.2.1 (R Core Team, 2017), using the lme4 package (Bates, Kliegl, Vasishth, & Baayen, 2015).

## 3 RESULTS

### 3.1 The effect of WFS on seed set of strawberry fruits

**Figure 1:**
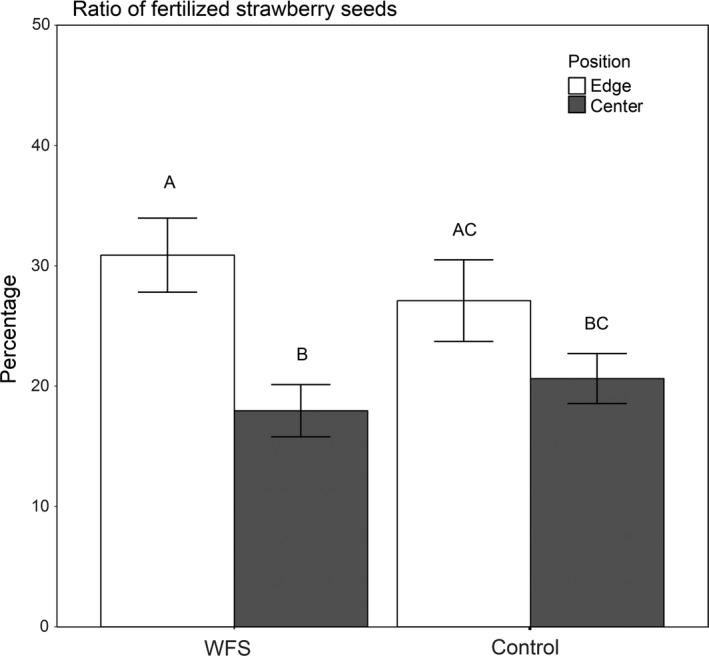
The ratio of fertilized strawberry seeds dependent on the treatment (fields with neighboring wildflower strips (WFS) and without (Control) and the location of sampling (crop edge, crop center). Different letters represent significantly different means at *p* ≤ 0.05

Seed set (proportion of fertilized seeds per fruit) of a total of 282 strawberry fruits across 2 years was analyzed (201 fruits from fields with and 81 from fields without neighboring WFS). Especially in 2016 high precipitation and flooding lead to a low number of developed fruits (*n* = 78). There was no overall difference of seed set between WFS and the control fields (*p* = 0.096, Table 1; Figure 1). However, a significantly higher proportion of fertilized seeds occurred at the edge compared to the center of the crop field (*p* = 0.004, Table 1; Figure 1), but only on the crop field with WFS, indicated by a significant interaction between WFS presence (field with WFS or control field without WFS) and within‐field location (edge or center) of plants (*p* = 0.047, Table 1; Suppoering Information Figure S2).

**Table 1:** Generalized linear mixed‐effects model showing the effect of management (wildflower strip [WFS] vs. control), location of sampled plants within the strawberry crop field (center and adjacent to WFS) and the interaction between management and location on the ratio of fertilized seeds of strawberry fruits (*n* = 205)

	Ratio of fertilized seeds			
	Estimate	*Z*	*SE*	*p*
(Intercept)	−1.53	−2.57	0.61	**0.011**
Management	0.56	1.66	0.34	0.096
Location	0.61	2.85	0.21	**0.004**
Management: location	−0.62	−1.91	0.33	**0.047**

Notes

Random effects: 1|year/site id/plant. Significant predictors (*p* < 0.05) are shown in bold.

### 3.2 The effect of WFS on pollinator communities

During the course of the study (27 hr of observation time), a total of 790 flower visits by insects were recorded (346 to WFS plants and 444 to strawberry crop plants). Of those, 197 visits in WFS (172 in strawberry) were from honey bees, 55 visits in WFS (130 in strawberry) from bumble bees (*Bombus* ssp.), 66 in WFS (50 in strawberry) from other wild bees and 87 in WFS (46 in strawberry) from hover flies. Flower visits from a total of 37 pollinator species (*A. *mellifera, six *Bombus ssp*., 20 other wild bee species, 10 hover fly species) were observed in WFS, which comprised a higher number of species per sampling round (6.33 ± 2.33) than in adjacent strawberry crops (2.82 ± 0.81), whereas honey bees, the hover fly *Eristalix tenax*, the bumble bee *B. terrestris,* and the wild bee *O. bicornis*, all generalist species, represented 94% of the pollinator community visiting strawberry crops. In total 12.5% of the species foraged in both, WFS and strawberry crops. Total visits of pollinators were higher in WFS (14.74 ± 2.26) than in adjacent strawberry fields (5.48 ± 0.61, ANOVA: *F*_1,106_, *p* < 0.001). However, there were no significant differences between crop edges to WFS (6.07 ± 1.17), crop center (3.52 ± 0.72) and crop edges to grassy strips (5.15 ± 0.99, ANOVA: *F*_2,78_, *p* = 0.187). Both, floral abundance (12.76 ± 3.60) and species richness of plants in sampled quadrats (6.74 ± 0.31) were higher in WFS than in adjacent strawberry crops (4.23 ± 0.91, *p* < 0.001, Table S4).

**Figure 2:**
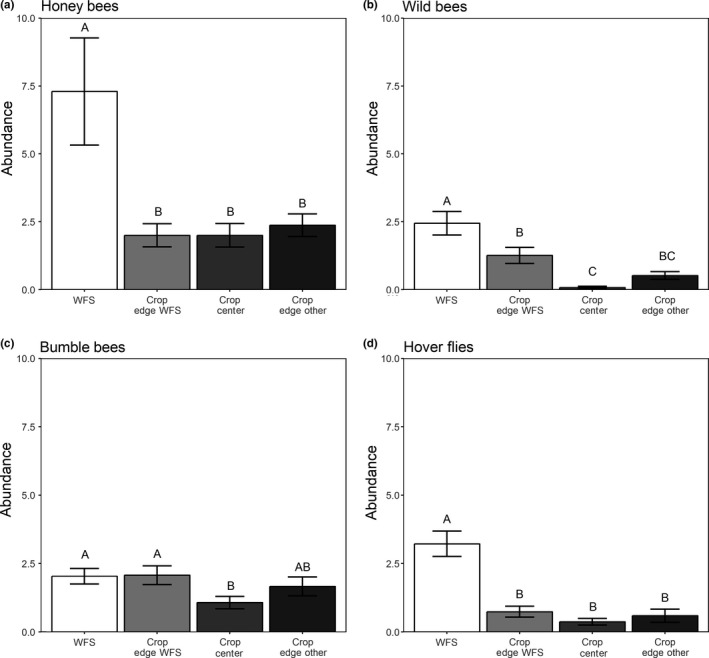
Abundance of the flower visitor groups (a) honey bees, (b) wild bees, (c) bumble bees, and (d) hover flies dependent on the location of sampling (WFS = wildflower strip, crop edge adjacent to WFS, crop center, crop edge to other habitat different to WFS). Different letters represent significantly different means at *p* ≤ 0.05

Wildflower strips supported higher flower visits of honey bees, wild bees (except bumble bees) and hover flies compared to adjacent strawberry crop fields, irrespective of the sampling location within the strawberry field (Table 2a; Figure 2). Wild bee visitation was enhanced in adjacent strawberry crops near WFS compared to field centers (Tukey's post hoc: *p* = 0.014), and intermediate at field edges near grassy strips (Tukey's post hoc: *p* = 0.001). The number of flower visits of bumble bees, however, did not differ between WFS and strawberry fields, except the field center, where it was significantly reduced compared to the WFS (*p* = 0.005, Table 2a; Figure 2). Flower abundance was a strong predictor for all studied taxa of pollinating insects (Table 2b). Thus, accounting for it, effects for the different groups changed, suggesting that the WFS effect is caused by the higher floral abundance. In case of wild bee and hover fly flower visits, the strong flower abundance effect lessened the location effect, which resulted in significant relationships between the center and the WFS (Table 2b; Figure 1). On the other hand, after accounting for floral abundance, the number of visits of honey bee and bumble bee was even higher in the crops compared to WFS, indicating that these taxa preferred foraging in strawberry fields/on strawberry flowers and were evenly distributed throughout the crop (Table 2b; Figure 1). However, species richness responses to field location differed among groups of pollinators (Table 2c). Species richness of wild bees excluding bumble bees and hover flies was lower in the strawberry crop than in WFS, whereas bumble bee species richness was only reduced in the center of the strawberry crop (*p* = 0.026, Table 2c).

**Table 2:** Generalized linear mixed‐effects models showing the effects of the location of sampling (WFS =wildflower strip (intercept), crop edge WFS, crop center, crop edge other) without (a), with flower units (b), and (c) species richness of flower‐visitor groups (Honey bees, bumble bees, wild bees, hover flies)

	Honey bees					Bumble bees					Wild bees					Hover flies				
	Estimate	*Z*	*SE*	*p*		Estimate	*Z*	*SE*	*p*		Estimate	*Z*	*SE*	*p*		Estimate	*Z*	*SE*	*p*	
Response: abundance																				
(a)																				
(Intercept)	1.04	3.62	0.3	**<0.001**	o	0.57	3.29	0.2	**<0.001**	n	0.63	3.01	0.2	**0.003**	n	0.94	4.71	0.2	**<0.001**	n
Crop edge WFS	−0.92	−2.4	0.4	**0.02**	o	0.02	0.10	0.2	0.923	n	−0.70	−2.72	0.2	**0.006**	n	−1.57	−4.97	0.3	**<0.001**	n
Crop center	−0.93	−2.5	0.4	**0.01**	o	−0.64	−2.83	0.2	**0.01**	n	−3.53	−4.82	0.7	**<0.001**	n	−2.26	−5.82	0.4	**<0.001**	n
Crop edge other	−0.67	−1.8	0.4	**0.07**	o	−0.20	−1.01	0.2	0.311	n	−1.59	−4.81	0.3	**<0.001**	n	−1.84	−5.31	0.3	**<0.001**	n
(b)																				
(Intercept)	−3.17	−5.72	0.6	**<0.001**	o	−2.03	−5.51	0.4	**<0.001**	n	−1.20	−9.81	0.2	**0.031**	n	−0.98	−1.72	0.6	**0.051**	o
Crop edge WFS	1.70	4.34	0.4	**<0.001**	o	1.66	6.15	0.3	**<0.001**	n	0.43	4.8	0.3	0.261	n	−0.39	−0.92	0.4	0.355	o
Crop center	1.68	4.32	0.4	**<0.001**	o	1.01	3.35	0.3	**<0.001**	n	−2.41	−2.06	0.7	**0.002**	n	−1.09	−2.3	0.5	**0.021**	o
Crop edge other	2.04	5.08	0.4	**<0.001**	o	1.50	5.28	0.3	**<0.001**	n	−0.42	1.3	0.3	0.326	n	−0.62	−1.43	0.4	0.153	o
Flower units	0.33	9.54	0.0	**<0.001**	o	0.19	8.72	0.1	**<0.001**	n	0.14	46.9	0.0	**<0.001**	n	0.15	3.78	0.0	**<0.001**	o
Response: species richness																				
(c)																				
(Intercept)						0.19	1.04	0.2	0.300	o	0.46	2.51	0.2	**0.012**	n	0.79	5.47	0.2	**<0.001**	n
Crop edge WFS						−0.06	−0.34	0.3	0.803	o	−0.83	−3.16	0.3	**0.002**	n	−1.64	−5.21	0.3	**<0.001**	n
Crop center						−0.66	−2.23	0.3	**0.031**	o	−3.18	−4.42	0.7	**<0.001**	n	−2.05	−5.54	0.4	**<0.001**	n
Crop edge other						−0.41	−1.52	0.3	0.141	o	−1.39	−4.31	0.3	**<0.001**	n	−1.93	−5.42	0.4	**<0.001**	n

Significant *p*‐values (<0.05) are shown in bold. n, models without overdispersion; o, models which included an observation‐level random factor.

**Figure 3:**
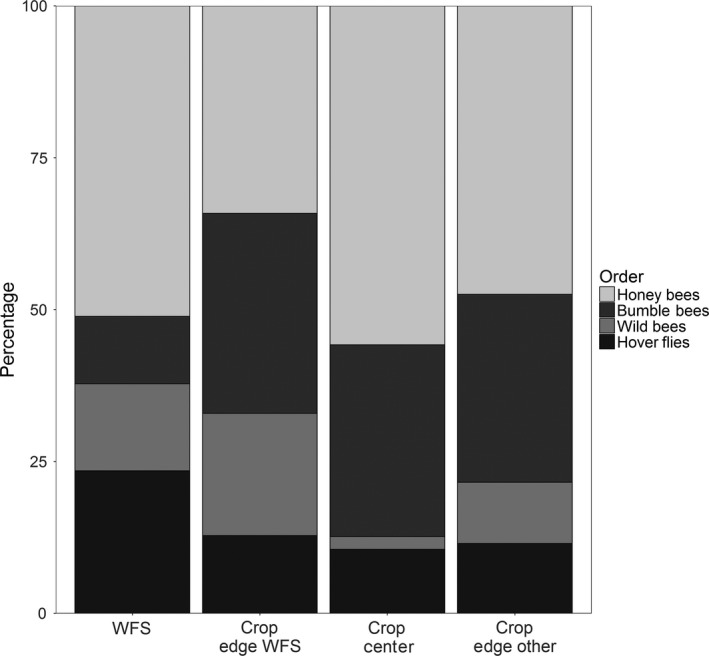
Proportion of pollinator groups (honey bees, bumble bees, wild bees, hover flies) within the pollinator community, dependent on the location of sampling (WFS = wildflower strip, crop edge adjacent to WFS, crop center, crop edge to other habitat different to WFS)

Community composition of strawberry flower visitors varied with distance to the WFS (significant flower visitor taxa ×distance interactions, Supporting Information Table S5; Figure 3). The average proportion of honey bees was higher in WFS (51%, Figure 3) than at the WFS adjacent edge of strawberry fields (34%; *p* = 0.019, Supporting Information Table S5; Figure 3), but not significantly different to other locations. Irrespective of within‐field location, the proportion of bumble bees was significantly higher in strawberry crops (approximately 30% of total pollinator share) than in WFS (11%; Supporting Information Table S5; Figure 3). The proportion of wild bees at the edge of the crop close to WFSs was significantly higher than in the WFS (*p* < 0.001), but significantly lower in the center of the crop (*p* = 0.037), where wild bees were least common (2%). Additionally, the share of hover flies was significantly lower at the edge of the WFS compared to inside the WFS (*p* = 0.033).

## 4 DISCUSSION

Our findings show increased pollination services at the edge of WFS compared to locally reduced pollination services at the center, which resulted in no significant difference in seed set between WFS and control fields.

We found similar spatial patterns of pollination service delivery and those of major strawberry pollinator groups. In fact, both pollination services and strawberry flower visitation was increased near WFS compared to the crop center, but only visitation by wild bees, but not by managed honey bees or hover flies. These findings suggest that the enhanced pollination services near WFS may be driven by positive spillover of wild bees from WFS into adjacent strawberry crops resulting in a functional spillover. Unfortunately, we have no data of the pollinator community in strawberry fields without WFS due time constraints during the field season. Nevertheless, our results are in a line with previous studies suggesting that such spillover effects may differ across taxonomic or functional groups of insects (Albrecht, Duelli, Müller, Kleijn, & Schmid, 2007; Balzan et al., 2016; Blitzer et al., 2012). They may be spatially more restricted in pollinator groups such as small wild bees compared to other pollinators, such as honeybees, with different foraging strategies acting on larger scales (Albrecht et al., 2007; Albrecht, Ramis, & Traveset, 2016; Steffan‐Dewenter, Münzenberg, Bürger, Thies, & Tscharntke, 2002). Wild bees may benefit particularly much locally from floral enhancements offering adequate food and nesting sources (Kremen & M'Gonigle, 2015; Steffan‐Dewenter et al., 2002). Our study suggests that these patterns have important functional implications for the provisioning of pollination services that should be considered when designing and implementation floral enhancement schemes aimed at promoting crop pollination services.

Interestingly, both flower visitation by wild bees and strawberry average seed set tended also to be higher at crop edges near grassy strip compared to crop centers, but increases were not significantly different but rather intermediate between measures near WFS and those in field centers (Figures 1 and 2). This suggests that even grassy strips with low amounts of flowering species influence the landscape complexity and that edge proximity rather than quality may matters for predicting pollinator abundance in a field.

In general, more flower visitors comprising a pronouncedly higher number of species were recorded in WFS rather than in adjacent strawberry crops. In fact, only 12.5% of the species foraged in both, WFS and strawberry crops, mainly common generalist species. Higher flower visits and species richness of pollinators in WFS were expected as they are designed to promote not only a few key crop pollinator species but rather to simultaneously promote diverse pollinator communities through the provision of a high diversity of flowering plant taxa offering complementary types of floral resources (pollen and nectar) continuously during the season (M'Gonigle, Williams, Lonsdorf, & Kremen, 2017; Tschumi, Albrecht, Entling, & Jacot, 2015). Also mass‐flowering crops can be highly attractive for many generalist species (Kleijn et al., 2015; Magrach et al., 2018). Indeed, although there were no hives of managed honey bees in the direct vicinity of the focal fields in our study, they were the most abundant group of strawberry visitors and preferred the crop over the WFS. Moreover, bumble bees showed no clear preference for the flower diverse WFS. This is in line with studies observing bumble bees which preferred mass resources like oilseed rape over flower‐diverse habitats (Westphal, Steffan‐Dewenter, & Tscharntke, 2003). Both groups, honey bees and bumble bees, were evenly distributed throughout the crop, while hover flies and wild bees were less abundant in field centers but found in similar densities at crop edges. This would infer that eusocial taxa preferentially forage on mass‐flowering crops (when in bloom) and are able to penetrate deep into agricultural fields. In contrast, wild bees and hover flies were more restricted to edge habitats and showed small‐scale spillover with WFS. Therefore, we suggest that species with a generalized use of food plants, like honey bees (Mitchell, Flanagan, Brown, Waser, & Karron, 2009) or bumble bees (Waser, Chittka, Price, Williams, & Ollerton, 1996) may be less dependent on flower diversity and more likely are not concentrated on WFS. Nevertheless, we have no data on the post‐crop period (June‐September), when there may be a dearth of floral resources and insects may change habitat preferences.

It is important to note that, despite the fact a diverse community of pollinators consumed flower resources offered by the WFS and the increased wild bee visitation and pollination of strawberry crops near WFS, the annual type of WFS studied here may not be effective in enhancing pollinator populations and their persistence in agricultural landscapes in the long term (>2 years). Perennial floral enhancement measures such as perennial WFS (e.g. Blaauw & Isaacs, 2014; Sutter et al., 2017) or hedgerow restoration schemes targeted on the floral food and nesting resource requirements of pollinators (e.g. Kremen & M'Gonigle, 2015) providing pollinators with floral resources and potentially important nesting and overwintering habitat over many years may be more effective measures to promote pollinator population growth and persistence in agroecosystems (M'Gonigle, Ponisio, Cutler, & Kremen, 2015). Moreover, positive effects on crop yield and economic benefits for farmers may become significant only after several years after the establishment of floral enhancements (Blaauw & Isaacs, 2014). Nevertheless, our study shows that even annual WFS can improve floral resource availability for diverse pollinator communities and increase pollination service to crops near WFS. Floral enhancement schemes including annual WFS should, however, ensure that floral resource provision to the local pollinator community is guaranteed by sowing strips each year within a geographically restricted area of the farmland. That includes permanent habitats offering suitable nesting and overwintering sites for pollinators, considering the often limited foraging distances of many pollinator taxa.

## 5 CONCLUSIONS AND MANAGEMENT IMPLICATIONS

We conclude that flowering plant species rich WFS can host wild bee diversity and promote pollination services in nearby crops, but that the latter may be spatially restricted to the crop edges near WFS. Thus, our results should encourage farmers to establish WFS on farmland to offer crop and non‐crop pollinators also outside the flowering period of mass‐flowering crops with floral resources throughout the season. Our study can therefore dispel concerns by farmers that floral enhancements such as WFS could reduce rather than increase crop pollination services to nearby crops by drawing crop pollinators, such as honey bees, away from them. Moreover, our findings indicate positive spillover effects from WFS into adjacent strawberry crops for wild bees, but not hover flies or managed honey bees, thereby resulting in shifts in crop pollinator communities in adjacent crops with potential consequences on crop pollination across the edge and central parts of crop fields. However, as this occurs at small spatial scales, there is a need for more diversified farming systems. This needs to be considered when designing and implementing floral enhancement measures. Based on these findings, establishing WFS in the center of crop fields should be a promising measure to maximize pollination benefits across fields, but further research integrating land opportunity costs in economic cost‐benefit analyses is required to further improve management guidelines for farmers regarding the optimal spatial arrangement of WFS.

## AUTHORS’ CONTRIBUTIONS

E.K., M.A., and D.G. conceived the ideas and/or designed methodology; D.G. and B.M. collected the data; D.G. and E.K. analyzed the data; D.G. led the writing of the manuscript. All authors contributed critically to the drafts and gave final approval for publication.

## DATA ACCESSIBILITY

Data available from the Dryad Digital Repository: https://doi.org/10.5061/dryad.0js540s
