# Supplementary material for: Wildflower strips enhance pollination in adjacent strawberry crops at the small scale
Source: Ecol Evol. 2018 Nov 6;8(23):11775–84. doi: 10.1002/ece3.4631 (PMC6303775; doi:10.1002/ece3.4631)
Supplement: Supplementary file 1 [file ECE3-8-11775-s001.docx]

**
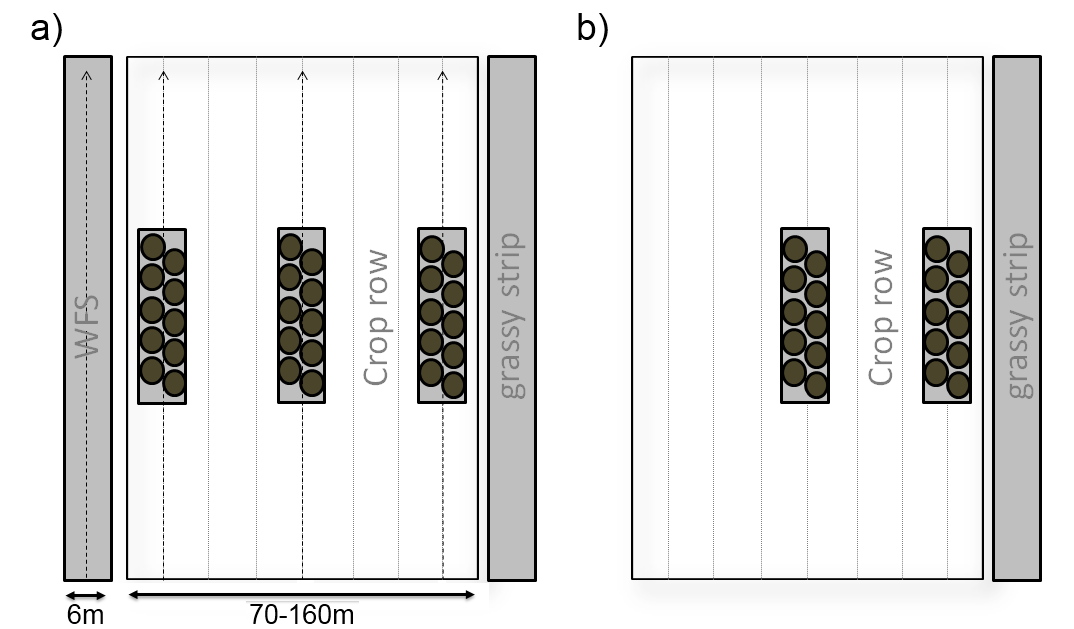
**

**Figure S1.** Schematic study design of a) crop fields with adjacent WFS and grassy strips, and b) crop fields without adjacent WFS (control). Potted Strawberry plants (n=10) were placed in the centre of the crop field, at the edge adjacent to grassy strips and in treatments with WFS (a) close to the WFS. Dotted arrows indicate walked belt transects, but only in treatments with WFS.

**
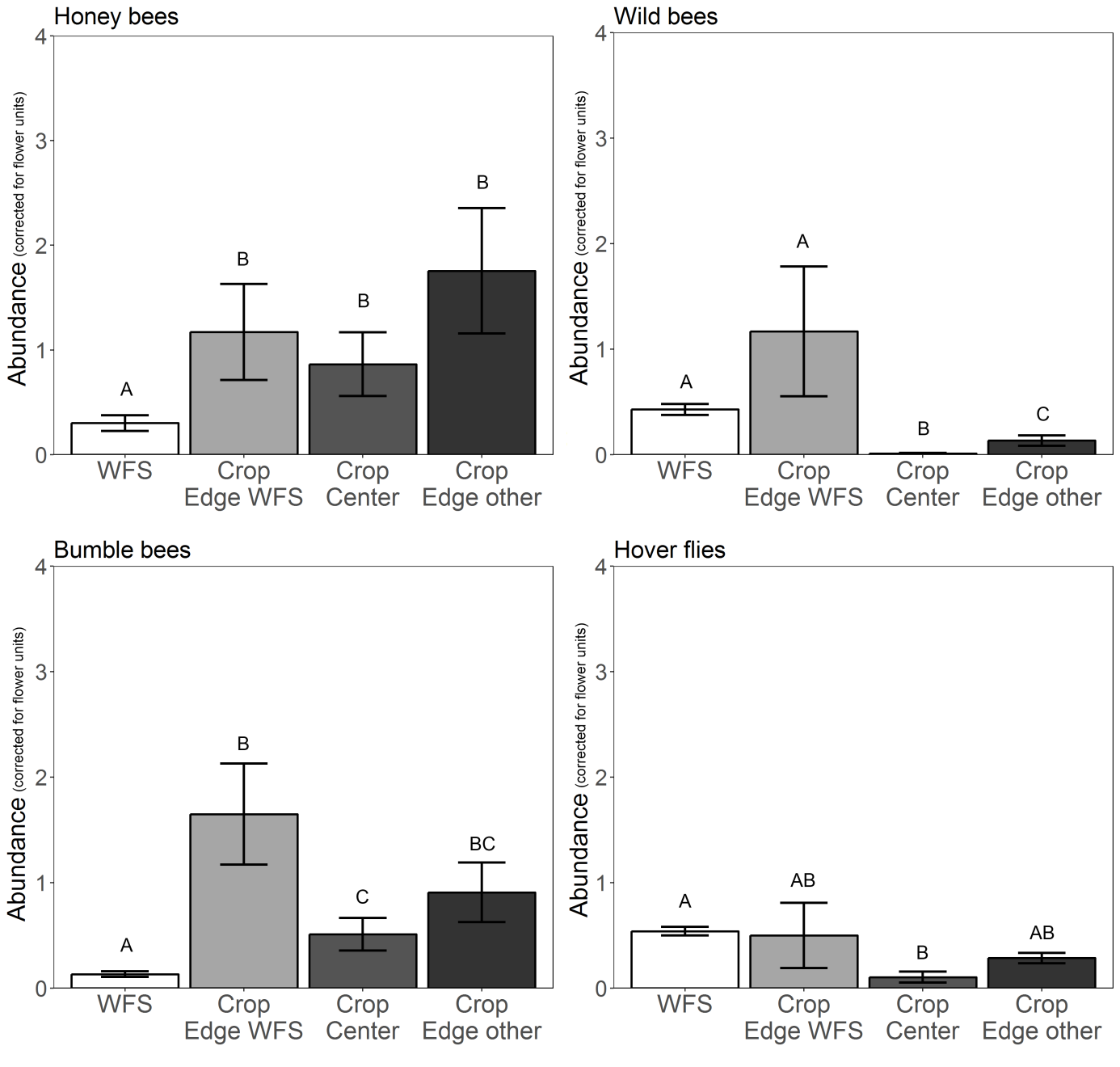
Figure S2.** Abundance of the flower visitor groups (a) honey bees, (b) wild bees, (c) bumble bees, and (d) hover flies corrected for flower units dependent on the location of sampling (WFS) wildflower strip, crop edge adjacent to WFS, crop centre, crop edge to other habitat different to WFS).
