# Supplementary material for: Wildflower strips enhance pollination in adjacent strawberry crops at the small scale
Source: Ecol Evol. 2018 Nov 6;8(23):11775–84. doi: 10.1002/ece3.4631 (PMC6303775; doi:10.1002/ece3.4631)
Supplement: Supplementary file 2 [file ECE3-8-11775-s002.docx]

**Supporting Information**

**Table S1.** Farm characteristics used for sampling in 2016 and 2017: Wildflower strips (WFS). ZH = Zürich, BE = Bern, SO = Solothurn, AG = Aargau. Three plots were only used in 2015 and three plots were resown in 2016 at the same plots. Landscape complexity and composition was measured within 500m around the fields (ratio of total landscape: SNH = semi-natural habitat; Agr_int = intensive agriculture).

| ID | Canton | Altitude (m) | Type | Flower mixture | Sowing dates (WFS) | Years of sampling | Size WFS (m2) | Distance crop centre (m) | Distance crop edge (m) | Forest (%) | SNH (%) | Agr_int (%) | Urban (%) |
| --- | --- | --- | --- | --- | --- | --- | --- | --- | --- | --- | --- | --- | --- |
| A15_RW | ZH | 522 | WFS | Mixture 1 | 09/2015 | 2016 | 1242 | 50 | 100 | 13.46 | 6.23 | 53.88 | 16.56 |
| A15_PN | BE | 443 | WFS | Mixture 1 | 09/2015 | 2016 | 2019 | 80 | 160 | 15.73 | 11.92 | 36.17 | 34.34 |
| A15_HS | ZH | 418 | WFS | Mixture 1 | 09/2015 | 2016 | 1248 | 40 | 80 | 7.89 | 11.13 | 56.64 | 23.39 |
| A15_UW | SO | 485 | WFS | Mixture 1;2 | 10/2015 ; 09/2016 | 2016 2017 | 1041 ; 1379 | 60 | 120 | 36.11 | 7.51 | 39.72 | 16.63 |
| A15_HL | AG | 436 | WFS | Mixture 1;2 | 09/2015 ; 09/2016 | 2016 2017 | 360 ; 911 | 45 | 90 | 11.08 | 7.22 | 42.21 | 39.04 |
| A15_MM | BE | 828 | WFS | Mixture 1;2 | 10/2015 ; 09/2016 | 2016 2017 | 1245 ; 1245 | 40 | 75 | 13.16 | 7.67 | 63.81 | 5.41 |
| P16_AG | SO | 424 | WFS | Mixture 2 | 04/2016 | 2016 2017 | 976 | 50 | 100 | 12.07 | 11.23 | 50.79 | 21.35 |
| P16_PN1 | BE | 443 | WFS | Mixture 2 | 04/2016 | 2016 2017 | 930 | 80 | 160 | 32.13 | 9.89 | 41.12 | 15.07 |
| P16_PN2 | BE | 431 | WFS | Mixture 2 | 04/2016 | 2016 2017 | 972 | 40 | 80 | 25.10 | 6.39 | 39.12 | 28.48 |
| P16_TR | BE | 539 | WFS | Mixture 2 | 04/2016 | 2016 2017 | 1514 | 45 | 90 | 28.12 | 9.20 | 42.01 | 20.65 |
| P16_UT | BE | 489 | WFS | Mixture 2 | 04/2016 | 2016 2017 | 1242 | 55 | 110 | 30.01 | 8.69 | 45.78 | 15.12 |
| P16_THK | BE | 728 | WFS | Mixture 2 | 04/2016 | 2016 2017 | 658 | 60 | 120 | 23.21 | 6.26 | 48.47 | 12.20 |
| C17_PN | BE | 445 | control | - | - | 2016 2017 | - | 80 | 160 | 21.07 | 5.82 | 31.23 | 33.30 |
| C17_SH | SO | 474 | control | - | - | 2016 2017 | - | 60 | 120 | 13.67 | 9.78 | 40.78 | 25.80 |
| C17_HR | ZH | 421 | control | - | - | 2016 2017 | - | 60 | 120 | 39.23 | 7.29 | 36.48 | 6.85 |
| C17_HS | ZH | 418 | control | - | - | 2016 2017 | - | 80 | 160 | 31.04 | 9.37 | 39.14 | 19.69 |
| C17_DBH | AG | 418 | control | - | - | 2016 2017 | - | 40 | 80 | 26.11 | 7.77 | 51.52 | 10.12 |
| C17_MW | BE | 429 | control | - | - | 2016 2017 | - | 35 | 70 | 26.22 | 8.06 | 45.56 | 16.12 |
| C17_MZ | BE | 557 | control | - | - | 2016 2017 | - | 50 | 100 | 11.32 | 10.69 | 48.47 | 19.62 |

**Table S2.** The different seed mixtures used in the WFSs in 2016 and 2017. Mixture 1 and 2 were sown in autumn 2015 (n=6) and 2016 (n=3), Mixture 2 in spring 2016 (n=6).

|  | Seed mixtures | | |
| --- | --- | --- | --- |
|  | Mixture 1 (2015) | Mixture 2 (2016) | Mixture 3 |
| *Anchusa arvensis* | x |  |  |
| *Anethum graveolens* | x | x |  |
| *Anthemis tinctoria* | x | x |  |
| *Camelina sativa* | x | x |  |
| *Campanula patula* | x |  | x |
| *Campanula rapunculoides* | x |  | x |
| *Centaurea scabiosa* | x |  | x |
| *Centaureus cyanus* | x | x | x |
| *Centaureus jacea* |  |  | x |
| *Cichorium intybus* | x |  | x |
| *Clinopodium vulgare* |  |  | x |
| *Consolida regalis* |  |  | x |
| *Crepis biennis* | x |  | x |
| *Crepis capillaris* |  |  | x |
| *Daucus carota* | x | x | x |
| *Echium vulgare* | x |  |  |
| *Fagopyrum esculentum* |  | x | x |
| *Hypochaeris radicata* | x |  | x |
| *Knautia arvensis* |  |  | x |
| *Lamium purpureum* |  | x | x |
| *Leontodon hispidus* | x |  |  |
| *Linum usitatissimum* | x |  | x |
| *Lotus corniculatus* | x |  |  |
| *Malva sylvestris* |  |  | x |
| *Melilotus albus* | x | x | x |
| *Onobrychis viciifolia* | x | x | x |
| *Origanum vulgare* |  |  | x |
| *Papaver rhoeas* | x | x | x |
| *Phacelia tanacetifolia* | x | x | x |
| *Picris hieracioides* | x | x |  |
| *Reseda lutea* | x |  | x |
| *Scabiosa columbaria* |  |  | x |
| *Sinapsis arvensis* | x | x | x |
| *Stachys annua* | x |  | x |
| *Stachys officinalis* |  |  | x |
| *Trifolium hybridum* | x |  |  |
| *Trifolium incarnatum* | x | x |  |
| *Trifolium pratense* | x | x | x |
| *Vicia sativa* |  | x |  |

n= 27 16 28

**Table S3.** Specimen sampled on the transects of the WFS and in the crops during flower visitor surveys.

| **Order** | **Family** | **Genus** | **Scientific name** | **WFS** | **Crop** | **Total** | **Specification** |
| --- | --- | --- | --- | --- | --- | --- | --- |
| Hymenoptera | Apidae | *Apis* | *Apis mellifera* | 197 | 172 | 369 | honey bee |
| Hymenoptera | Apidae | *Bombus* | *Bombus terrestris* | 16 | 99 | 115 | bumble bee |
| Hymenoptera | Apidae | *Bombus* | *Bombus lapidarius* | 13 | 31 | 44 | bumble bee |
| Hymenoptera | Apidae | *Bombus* | *Bombus pratorum* | 7 | 0 | 7 | bumble bee |
| Hymenoptera | Apidae | *Bombus* | *Bombus pascuorum* | 13 | 0 | 13 | bumble bee |
| Hymenoptera | Apidae | *Bombus* | *Bombus hortorum* | 5 | 0 | 5 | bumble bee |
| Hymenoptera | Apidae | *Bombus* | *Bombus barbutellus* | 1 | 0 | 1 | bumble bee |
| Hymenoptera | Apidae | *Osmia* | *Osmia bicornis* | 16 | 42 | 58 | wild bee |
| Hymenoptera | Apidae | *Osmia* | *Osmia caerulsecens* | 7 | 0 | 7 | wild bee |
| Hymenoptera | Apidae | *Lasioglossum* | *Lasioglossum laticeps* | 6 | 0 | 6 | wild bee |
| Hymenoptera | Apidae | *Lasioglossum* | *Lasioglossum malachurum* | 3 | 0 | 3 | wild bee |
| Hymenoptera | Apidae | *Lasioglossum* | *Lasioglossum politum* | 2 | 0 | 2 | wild bee |
| Hymenoptera | Apidae | *Lasioglossum* | *Lasioglossum pauxillum* | 1 | 0 | 1 | wild bee |
| Hymenoptera | Apidae | *Lasioglossum* | *Lasioglossum villosulum* | 3 | 0 | 3 | wild bee |
| Hymenoptera | Apidae | *Lasioglossum* | *Lasioglossum nigripes* | 2 | 0 | 2 | wild bee |
| Hymenoptera | Apidae | *Lasioglossum* | *Lasioglossum glabriusculum* | 3 | 0 | 3 | wild bee |
| Hymenoptera | Apidae | *Lasioglossum* | *Lasioglossum morio* | 3 | 0 | 3 | wild bee |
| Hymenoptera | Apidae | *Andrena* | *Andrena flavipes* | 4 | 8 | 12 | wild bee |
| Hymenoptera | Apidae | *Andrena* | *Andrena ovatula* | 2 | 0 | 2 | wild bee |
| Hymenoptera | Apidae | *Andrena* | *Andrena gravida* | 3 | 0 | 3 | wild bee |
| Hymenoptera | Apidae | *Andrena* | *Andrena lagopus* | 2 | 0 | 2 | wild bee |
| Hymenoptera | Apidae | *Halictus* | *Halictus tumulorum* | 1 | 0 | 1 | wild bee |
| Hymenoptera | Apidae | *Halictus* | *Halictus scabiosae* | 2 | 0 | 2 | wild bee |
| Hymenoptera | Apidae | *Halictus* | *Halictus simplex* | 1 | 0 | 1 | wild bee |
| Hymenoptera | Apidae | *Hylaeus* | *Hylaeus communis* | 2 | 0 | 2 | wild bee |
| Hymenoptera | Apidae | *Chelostoma* | *Chelostoma rapunculi* | 1 | 0 | 1 | wild bee |
| Hymenoptera | Apidae | *Colletes* | *Colletes similis* | 2 | 0 | 2 | wild bee |
| Diptera | Syrphidae | *Eristalis* | *Eristalis tenax* | 16 | 41 | 57 | hover fly |
| Diptera | Syrphidae | *Episyrphus* | *Episyrphus balteatus* | 15 | 0 | 15 | hover fly |
| Diptera | Syrphidae | *Merodon* | *Merodon equestris* | 5 | 5 | 10 | hover fly |
| Diptera | Syrphidae | *Syritta* | *Syritta pipiens* | 8 | 0 | 8 | hover fly |
| Diptera | Syrphidae | *Spgaerophoria* | *Sphaerophoria_scripta* | 14 | 0 | 14 | hover fly |
| Diptera | Syrphidae | *Melanostoma* | *Melanostoma scalare* | 7 | 0 | 7 | hover fly |
| Diptera | Syrphidae | *Melanostoma* | *Melanostoma mellinum* | 14 | 0 | 14 | hover fly |
| Diptera | Syrphidae | *Eristalis* | *Eristalis arbustorum* | 6 | 0 | 6 | hover fly |
| Diptera | Syrphidae | *Eumerus* | *Eumerus strigatus* | 1 | 0 | 1 | hover fly |
| Diptera | Syrphidae | *Eupeodes* | *Eupeodes corollae* | 1 | 0 | 1 | hover fly |

**Table S4.** Mean values (+SEs) of flower units; flower species richness and seed set of strawberry fruits for a) the different locations on the plots (WFS= wildflower strip; crop edge with adjacent WFS; crop centre on WFS plots; crop edge on WFS plots with adjacent grass stripe; crop centre on control plots; crop edges on control plots with adjacent crass stripe) and b) the different seed mixtures used. Significant p-values (<0.05) are shown in bold.

| Variable | Flower abundance | |  | Flower species richness | |  | Fertilized strawberry seeds | |
| --- | --- | --- | --- | --- | --- | --- | --- | --- |
|  | Mean | SE |  | Mean | SE |  | Mean | SE |
| *a) Location* |  |  |  |  |  |  |  |  |
| WFS | **12.76** | 2.60 |  | **6.74** | 0.31 |  | - | |
| Crop edge WFS | 4.42 | 0.56 |  | 1.00 | 0.00 |  | 0.31 | 0.03 |
| Crop centre WFS | 4.39 | 0.70 |  | 1.00 | 0.00 |  | **0.18** | 0.02 |
| Crop edge other | 4.24 | 1.47 |  | 1.00 | 0.00 |  | 0.24 | 0.02 |
| Crop centre (control) | 4.89 | 1.01 |  | 1.00 | 0.00 |  | **0.21** | 0.02 |
| Crop edge other (control) | 4.76 | 0.87 |  | 1.00 | 0.00 |  | 0.27 | 0.03 |
|  |  |  |  |  |  |  |  |  |
| *b) Seed mixtures* |  |  |  |  |  |  |  |  |
| mixture 1 | 13.27 | 1.97 |  | 7.28 | 0.34 |  | 0.25 | 0.02 |
| mixture 2 | 12.48 | 1.42 |  | 6.91 | 0.27 |  | 0.32 | 0.04 |
| mixture 3 | 13.71 | 2.76 |  | 6.74 | 0.16 |  | 0.24 | 0.02 |

**Table S5.** Generalized linear mixed effects models showing the effects of the flower visitor group (honey bees (intercept), bumblebees, wild bees, hoverflies), location of sampling (WFS (intercept), edge to WFS, centre of the crop, edge of the crop) and possible interactions of both. Significant p-values (<0.05) are shown in bold.

|  |  |  |  |  |  |
| --- | --- | --- | --- | --- | --- |
|  |  | Ratio | | | |
|  |  | Estimate | Z | SE | P |
| (Intercept) |  | 0.28 | 2.57 | 0.11 | **0.01** |
| Bumble bees |  | -2.24 | -11.43 | 0.2 | **<.001** |
| Wild bees |  | -2.11 | -11.10 | 0.19 | **<.001** |
| Hover flies |  | -1.74 | -9.94 | 0.18 | **<.001** |
| Crop edge WFS |  | -1.2 | -5.63 | 0.21 | **<.001** |
| Crop centre |  | -0.28 | -1.15 | 0.24 | 0.252 |
| Crop edge other |  | -0.51 | -2.34 | 0.22 | **0.019** |
| Bumble bees:Crop edge WFS |  | 2.17 | 6.67 | 0.33 | **<.001** |
| Wild bees:Crop edge WFS |  | 1.66 | 4.98 | 0.33 | **<.001** |
| Hover flies:Crop edge WFS |  | 0.75 | 2.13 | 0.35 | **0.033** |
| Bumble bees:Crop centre |  | 1.08 | 2.76 | 0.39 | **0.006** |
| Wild bees:Crop centre |  | -1.61 | -2.08 | 0.77 | **0.037** |
| Hover flies:Crop centre |  | -0.38 | -0.84 | 0.45 | 0.402 |
| Bumble bees:Crop edge other |  | 1.24 | 3.48 | 0.36 | **<.001** |
| Wild bees:Crop edge other |  | 0.23 | 0.57 | 0.41 | 0.572 |
| Hover flies:Crop edge other |  | -0.04 | -0.11 | 0.39 | 0.916 |
